# Temperature Changes in Primary and Permanent Teeth Dentine of Varying Thicknesses Following Irradiation by Two Light Curing Units

**DOI:** 10.7759/cureus.44029

**Published:** 2023-08-24

**Authors:** Reem Alfares, Amani Agha, Obada Jabbour

**Affiliations:** 1 Pediatric Dentistry, University of Hama, Hama, SYR; 2 Barts and the London School of Medicine and Dentistry, Queen Mary University of London, London, GBR

**Keywords:** primary teeth, dentin thickness, temperature rise, led, halogen light

## Abstract

Aim: This study was performed to determine the temperature rise under human dentin discs of different thicknesses from primary and permanent teeth during the photo-curing process using quartz tungsten halogen (QTH) or light-emitting diode (LED).

Materials and methods: The current experimental study sample consisted of 160 dentin discs of different thicknesses (0.5, 1, 1.5, and 2 mm), of which 80 dentin discs were prepared from sound lower second primary molars, and the remaining 80 dentin discs were prepared from sound lower third permanent molars extracted surgically for various reasons. A "K" type of thermal tentacle was placed in the center of an acrylic resin base, followed by the placement of a dentin disc. Then, the thermal changes were measured during the photo-curing of the composite using a second LED or QTH light curing unit for 20 s. Statistical evaluation was performed using the IBM SPSS Statistics® Version 20.0 software system (SPSS Inc., Chicago, IL, USA).

Results: The current study found that the temperature rise in primary teeth (1.17-2.96°C) is significantly lower compared to the rise in permanent teeth (1.55-3.33°C), regardless of the dentin disc thickness or light curing unit used. The temperature rise decreases significantly when the thickness of dentin discs increases, regardless of the type of teeth or light curing unit used (P<0.05). Furthermore, QTH causes less temperature rise (1.17-2.65°C) compared to LED (1.61-3.33°C).

Conclusions: The temperature rise during polymerization of the resin composite with the second-generation LED appeared to be below 5.5°C. Hence, it appears to be safe for use during the restoration of primary teeth. Primary teeth dentin might be more effective than permanent teeth dentin in protecting the dental pulp.

## Introduction

Heat generated within the tooth during operative procedures can cause thermal damage to the dental pulp. However, the extent of thermal trauma that can be tolerated by the dental pulp is unknown [1]. It was documented that the dental pulp can withstand small temperature changes of 5.5 ºC above normal temperature (37 ºC) for relatively short periods of time (10 s) without permanent damage. However, extreme temperature changes above 5.5 ºC or prolonged exposure to high temperatures might cause pulpal changes of differing degrees [2].

Due to its low thermal conductivity, dentin is considered to be an excellent thermal insulator [3]. Therefore, the temperature rise in the pulp chamber is greatly influenced by the thickness of residual dentin. Thus, as that thickness reduces, the temperature of the pulp chamber rises [4].

Quartz tungsten halogen (QTH) light curing units (LCUs) are one of the most commonly used LCUs to cure dental composite, but this technology has several drawbacks [5]. Halogen bulbs have a limited effective lifetime of around 40-100 hours [6]. Light filters degrade with time due to their proximity to the halogen bulb’s heat. This, subsequently, can reduce the efficiency of the light output. The basic principle of light conversion in QTH bulbs has proven to be inefficient, as the light power output is less than 1% of the consumed electrical power due to the wide spectral output [6].

Light-emitting diodes (LEDs) were introduced to the market to address the previously mentioned issues associated with conventional QTH technology. Since the spectral output of these blue LEDs primarily falls within the range of wavelengths that most dental composites' camphorquinone photoinitiators absorb (400-500 nm), LEDs don't need filters to produce blue light [7]. Furthermore, LEDs have an expected lifetime of several thousand hours (10,000 hours) without showing a significant degradation of light emission over time [8]. LEDs are resistant to shock and vibration, and since they require less power to operate than halogen lights, this makes them suitable for portable use [7].

Evidence suggested significant chemical and morphological differences between primary and permanent teeth [9,10]. Primary dentin's tubule diameter appears to be larger than permanent dentin's [9]. Moreover, Johnson [10] reported that the central region of coronal dentin in permanent teeth is considerably harder than dentin from the same region in primary teeth. Based on the observation that the degree of mineralization is positively correlated with hardness, it was assumed that permanent teeth have a higher degree of mineralization compared to primary teeth.

With the increasing use of composite resin in the restoration of primary teeth in the third millennium, it is important to select the correct LCU that will not cause harmful overheating of the pulp. In view of this concern, the purpose of this in vitro study is to compare the effect of LED and QTH light curing units and different dentin thicknesses on the temperature rise on both primary and permanent teeth experimentally. The null hypothesis of the present in vitro study is that the temperature rise occurring during light curing of composites would be the same regardless of the photo-curing method, dentin thickness, or type of teeth.

This article was previously posted to the ResearchSquare preprint server on October 24, 2022, https://doi.org/10.21203/rs.3.rs-2193200/v1.

## Materials and methods

This study was approved by the Research Ethics Committee of the Faculty of Dentistry-University of Hama, Hama, Syrian Arab Republic (No: 659-28/04/2021).

**Figure 1:**
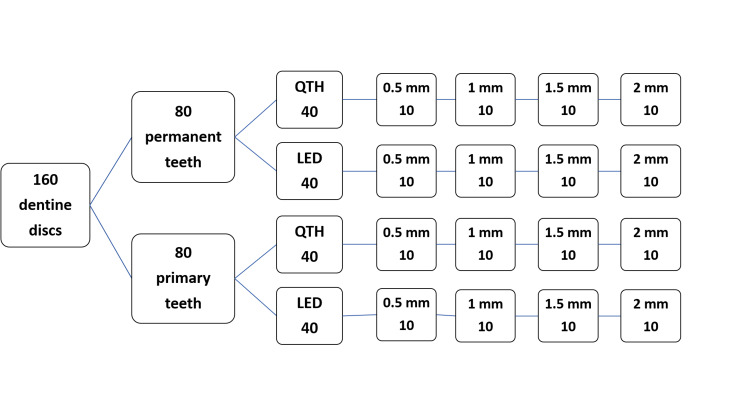
Distribution of the sample. QTH: quartz tungsten halogen, LED: light emitting diode.

**Figure 2:**
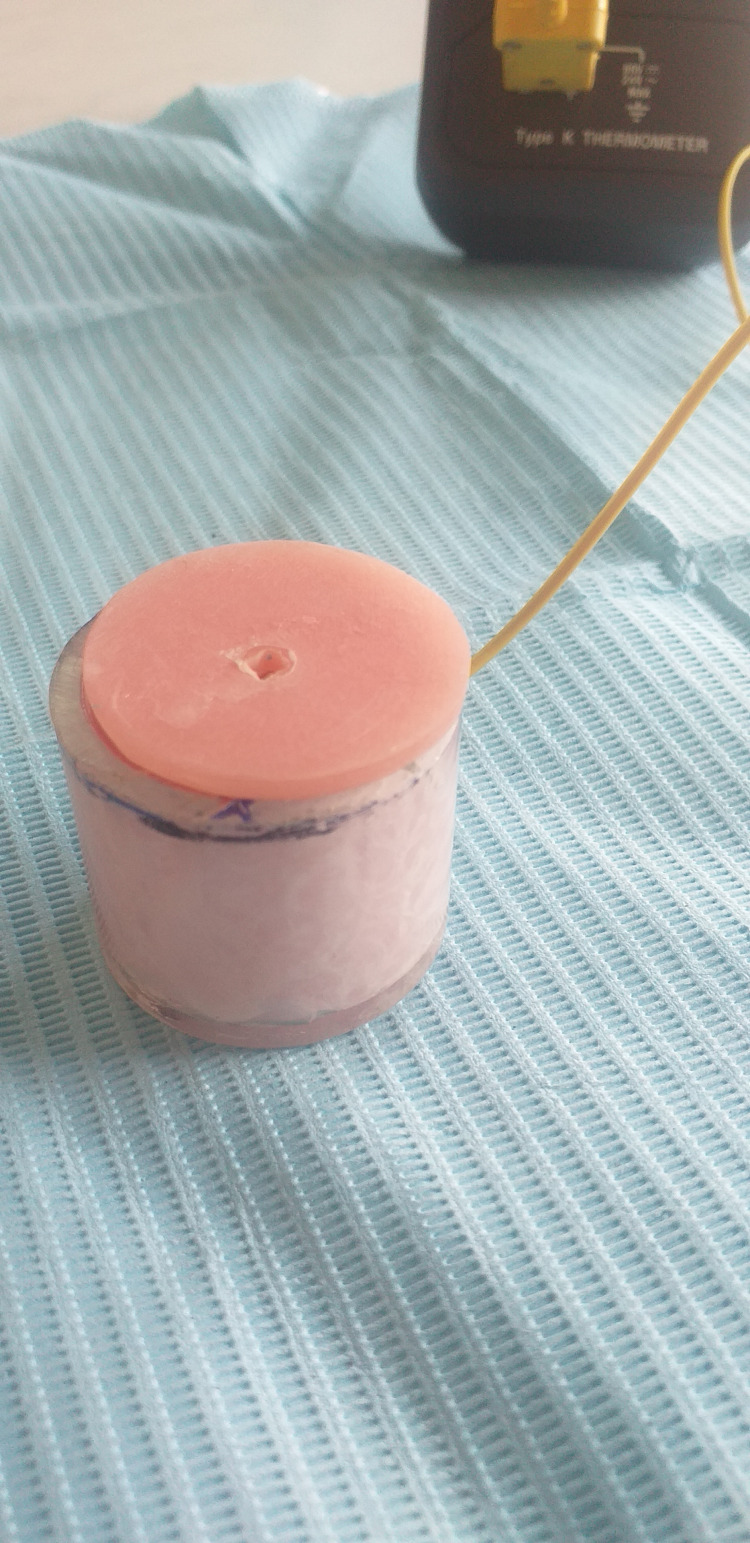
The acrylic base.

A total of 160 dentin discs, as can be seen in Figure 1, were used in this study. Sample size estimation was calculated using power and sample size calculation computer software (G*Power 3.1.9.7 Software, New York, USA). At α = 0.05 and with a power of 0.95, a minimum of 10 samples per group was required. Primary mandibular multiple root teeth freshly extracted for physiological root resorption reasons and third mandibular molars freshly extracted were cleaned with NaOcl-moistened gauze and stored in chloramine-T solution (1%) at 4 ºC for one week according to ISO/TS 11405. Subsequently, only the teeth without any presence of decay or any restorative materials were selected. Selected teeth were mounted on an acrylic block, leaving only the clinical crown exposed. Each acrylic block was sectioned perpendicular to the long axis of the mounted tooth (Figure 2). At that point, the thickness of the dentin disc was adjusted with wet carborundum paper and verified by a caliper. To maintain dentin moisture, blocks with mounted teeth were then stored in distilled water until they were used. Dentin discs used were also of variable thicknesses (0.5, 1, 1.5, and 2 mm) to mimic in vivo conditions.

**Figure 3:**
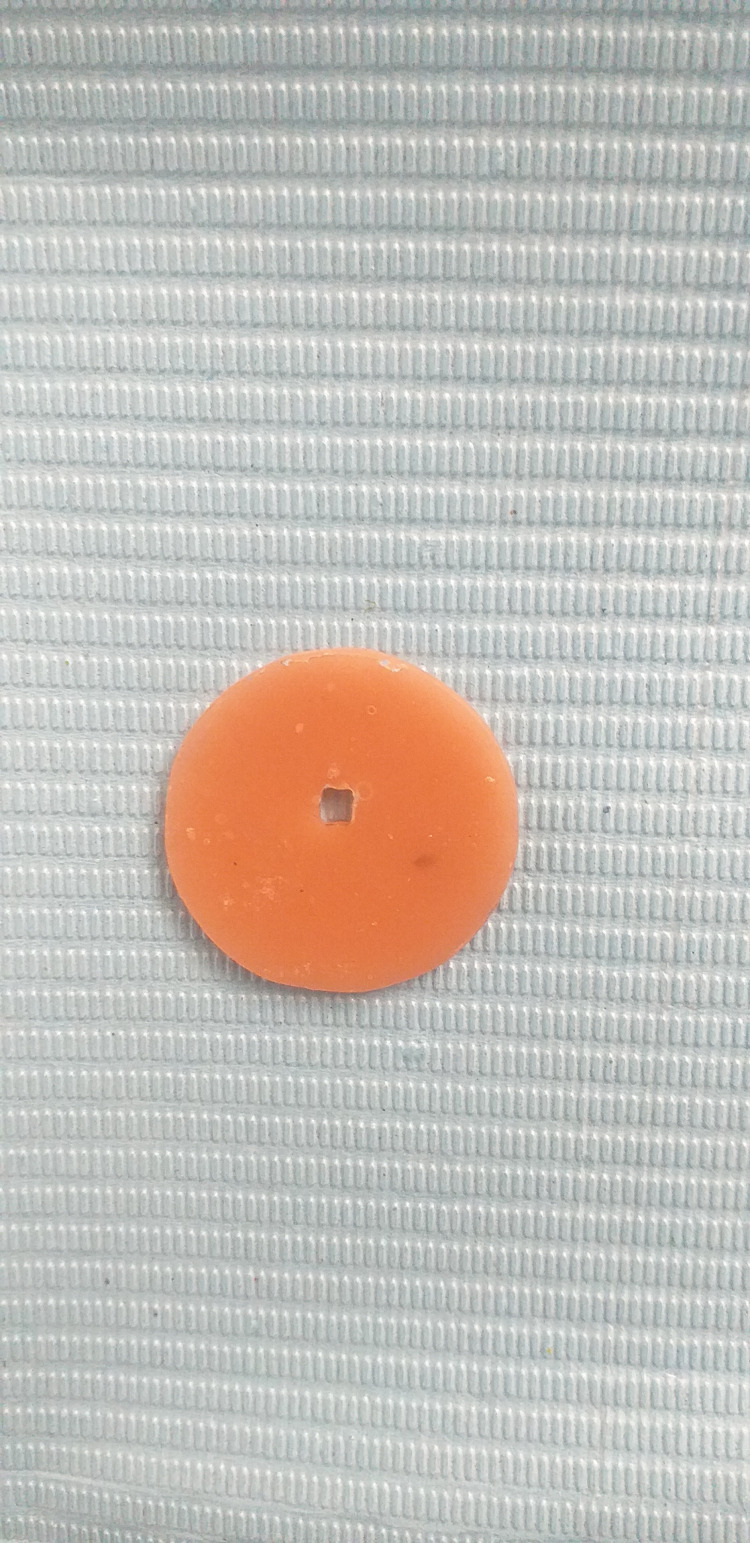
The acrylic disc.

A hybrid resin composite Z250 (3M-ESPE Dental Products, St. Paul, MN, USA) was used in 2 mm thickness, mimicking the clinical incremental use of this material. The thickness of the composite was standardized by an acrylic resin disc placed above the dentin disc (Figure 3). This disc had a central hole of 2 mm depth and a similar diameter to the diameter of the dentin disc. The resin composite was directly placed in this hole on the dentin disc.

**Figure 4:**
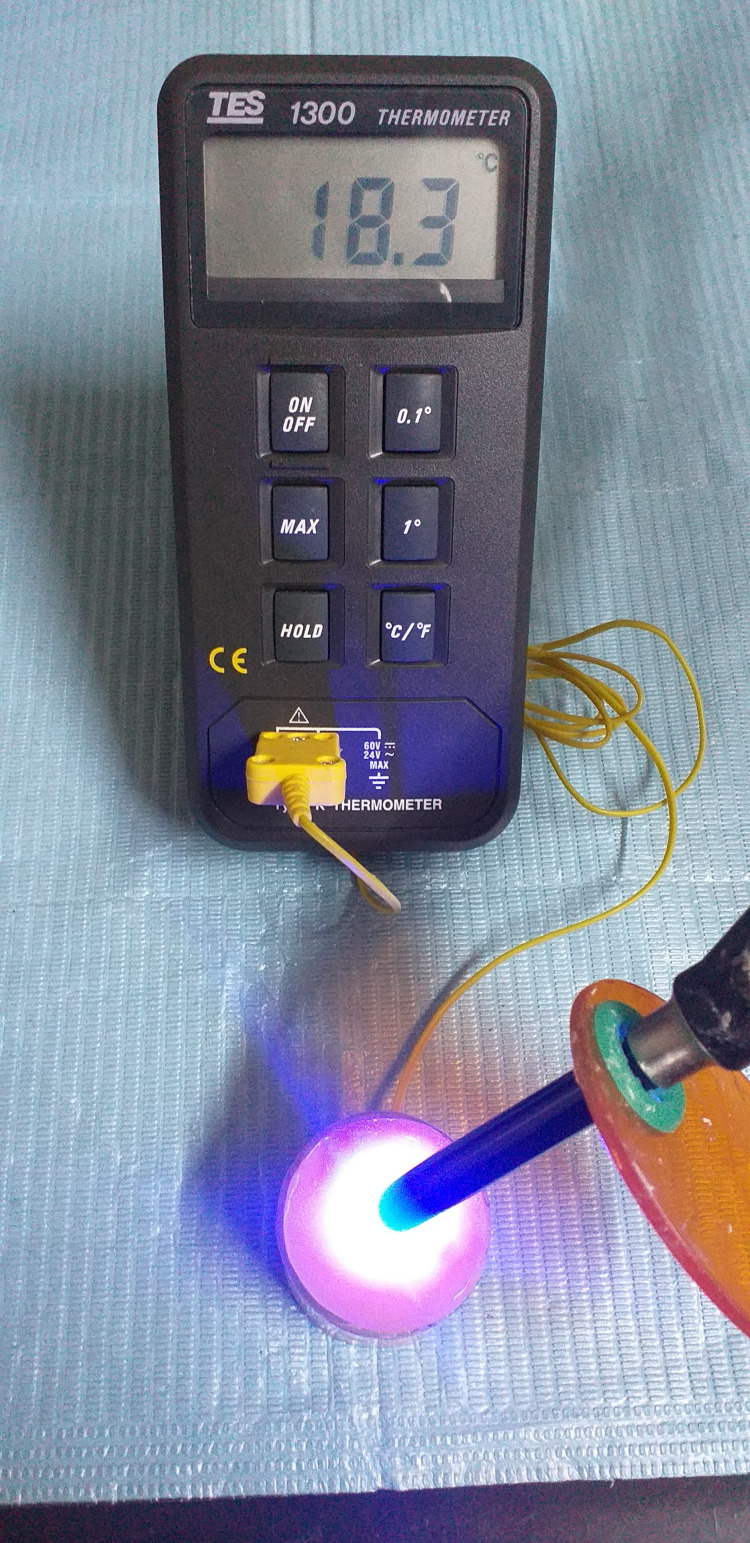
Irradiation of composite resin.

The composite material was covered with a Mylar strip (EHROS, Medeco, Chicago, IL) to prevent adhesion with the LCU tip. Then, it was irradiated from the top with direct contact (the distance was 0 mm) using one of the curing lights tested LED lamp Bluedent Smart (BG Light LTD, serial number: 019645) or the halogen lamp Chromalux 75 (serial number: 7047) (Table 1, Figure 4). The temperature was recorded using a K-type thermocouple (TES Digital Thermometer, serial number: 100408635), which was in direct contact with the dentin discs. All measurements were performed by the same investigator.

The light curing process was conducted according to the time recommended by the manufacturers of composites, and the temperature was recorded at the end of the procedure. The temperature rise was recorded at two levels: (1) baseline temperature following temperature stabilization (14±1°C) T1 and (2) maximum temperature during photo-polymerization of resin composite T2. To obtain the temperature rise ∆T, the baseline temperature was deducted from the maximum temperature.

**Table 1:** Details of the light curing units used in this study. QTH: quartz tungsten halogen, LED: light emitting diode, LCU: light curing units.

LCU	Intensity of light	Exposure time	Total energy
Chromalux 75 (QTH)	650-800 mW/cm^2^	20 s	130-160 J/cm^2^
Bluedent smart (LED)	800-1200 mW/cm^2^	20 s	200 J/cm^2^

Statistical evaluation

The results were analyzed using the IBM SPSS Statistics® Version 20.0 software system (SPSS Inc., Chicago, IL, USA). The Kruskal-Wallis and Mann-Whitney-U tests were used to evaluate the differences among the tested curing units and between permanent and primary teeth. The Mann-Whitney-U test was used to compare the mean temperature rise in each curing unit for different dentin thicknesses. The means were compared at the 5% significance level (p = 0.05).

## Results

**Figure 5:**
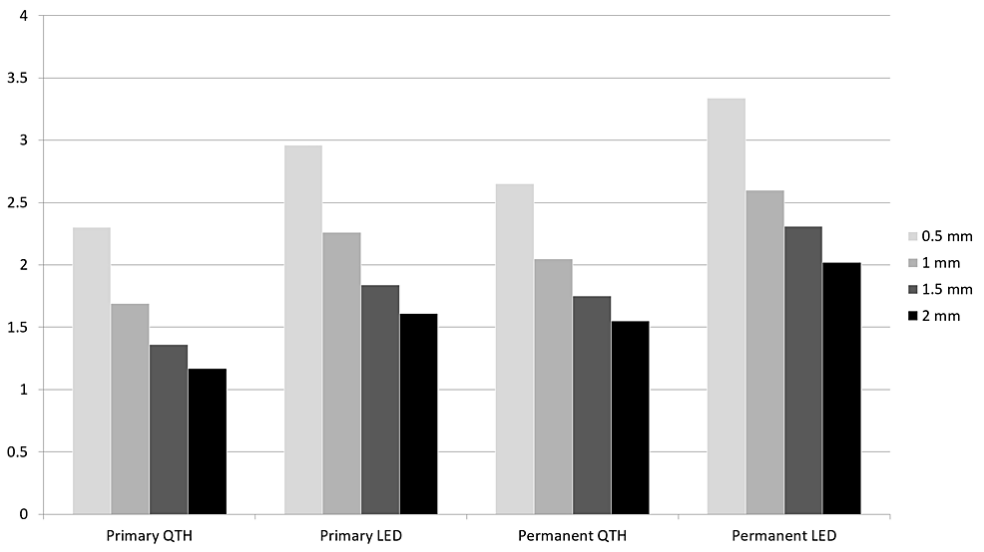
Results of temperature rise (°C) for the experimental groups. LED: light-emitting diode, QTH: quartz tungsten halogen.

The means and standard deviations of the temperature rise values of the dentin discs are listed in Table 2 and Figure 5. Kruskal-Wallis revealed significant differences in temperature rise values according to the type of LCU, dentin thickness, and type of teeth (p<0.05). The lowest values were recorded in primary teeth dentin with a thickness of 2 mm during resin composite polymerization with QTH (1.17°C), whereas resin composite cured with LED over permanent teeth dentin with a thickness of 0.5 mm exhibited the highest mean values in temperature rise (3.33°C).

**Table 2:** Mean temperature rise values (°C) for each dentin thickness and curing unit evaluated for each primary and permanent teeth. *Standard deviations are given in parentheses. QTH: quartz tungsten halogen, LED: light emitting diode.

Permanent teeth		Primary teeth		Thickness
LED	QTH	LED	QTH	
3.33 (0.75)	2.65 (0.44)	2.96 (0.49)	2.30 (0.40)	0.5 mm
2.60 (0.47)	2.05 (0.43)	2.26 (0.51)	1.69 (0.47)	1 mm
2.31 (0.69)	1.75 (0.52)	1.84 (0.45)	1.36 (0.40)	1.5 mm
2.02 (0.49)	1.55 (0.44)	1.61 (0.39)	1.17 (0.37)	2 mm

According to the results of Mann-Whitney-U tests, significant differences were observed among the tested LCU for all dentin thicknesses and types of teeth (permanent or primary) (p 0.05). The QTH curing unit caused less temperature rise than the LED unit, regardless of the dentin disc thickness or the type of dentition.

Examining differences between primary and permanent teeth revealed that primary teeth dentin produces a significantly lower temperature rise than permanent teeth dentin in the 1.5 and 2-mm-thick groups (p<0.05), while there was no significant difference between primary and permanent dentin in the 0.5 and 1-mm-thick groups (p>0.05).

An inverse proportion was found between the mean temperature rise values and the dentin thickness, regardless of the LCU tested or the type of teeth. In the primary teeth group, the 0.5-mm-thick group exhibited a significantly higher temperature rise than other thick groups (p<0.05). Significantly higher temperature rise values were recorded in the 1-mm-thick group than in the 1.5- and 2-mm-thick groups (p<0.05). However, there were no significant differences between dentin discs with thicknesses of 1 and 1.5 mm (p>0.05).

Polymerization of resin composite over permanent teeth dentin with a thickness of 0.5 mm produces a significantly higher temperature rise than dentin discs with thicknesses of 1, 1.5, and 2 mm (p<0.05). However, there were no significant differences between the other thickness groups (p>0.05).

## Discussion

In the present study, three different variables were studied: the temperature rise when curing by two different light curing units, the temperature rise through different dentin thicknesses, and the temperature rise through the dentin of permanent and primary teeth. The first null hypothesis of the present study has been rejected: that there were no significant differences in temperature rise between photo-curing methods due to the statistically significant difference observed in temperature rise between the LCU tested. Regarding the thickness of the dentin, a significantly inverse proportion was found between the mean temperature rise values and the dentin thickness regardless of the LCU tested and type of teeth; therefore, the second null hypothesis has been rejected. On the other hand, primary tooth dentin produces a significantly lower temperature rise than permanent tooth dentin in the 1.5 and 2-mm-thick groups, while there was no significant difference between primary and permanent dentin in two other thicknesses; therefore, the third null hypothesis has been partially rejected.

One storage media was used to eliminate any possible variation in deterioration. Therefore, variation in the thermal conductivity of dentin [11]. It is commonly believed that temperature increases cause irreversible damage to the pulp [2]. Zach and Cohen [2] studied the effects of heat on the pulp tissue of Macaca rhesus monkeys. The authors concluded that even a small increase of 5.5°C in intra-pulpal temperature can cause irreversible pulpitis in 15% of Macaca rhesus monkey teeth. The peak values recorded in this study were lower than 5.5°C in all conditions. Based on the results of the current study, it may be suggested that second-generation LED could be used safely in primary and permanent teeth as an alternative to QTH.

According to Loney and Price [12], a thicker dentin can reduce the temperature transferred to the pulp as a result of dentin’s low thermal conductibility, which agrees with this study. For all the light curing units and types of teeth included in this study, the increase of temperature through 2-mm-thick dentin was less pronounced than through 0.5-mm-thick dentin. This agrees with previous research, which confirmed that the remaining dentin thickness is inversely proportional to the temperature rise [4].

QTH light units have several drawbacks [12,13]. Compared to QTH lights, LEDs produce less heat from the light tip [14]. The spectral output of gallium nitride blue LEDs conveniently falls within the absorption spectrum of camphorquinone used in dental composites [15]. Therefore, these units do not require filters to produce blue light. LEDs are cordless because they are powered by rechargeable batteries [16]. The life span of LED curing units is about 10,000 hours with minimum performance wear, so it is significantly longer than the life span of QTH, which is about 100 hours [13,17].

Early reports of lower temperature rise associated with LEDs were attributed to first-generation LEDs with low power intensities (usually in the range of 400 mw/cm^2^ or even less) [18]. However, second-generation LEDs have higher power intensities (1000-2000 mw/cm^2^) [19]. The temperature rise caused by the light curing process depends not only on the power intensity but also on the total energy (total energy = power intensity × exposure time/1,000) produced by the light curing unit [6]. The QTH unit (Chromalux 75) used in this study emitted a total energy of 13-16 J/cm^2^ (650-800 mw/cm^2^ for 20 s). Under similar conditions, the LED unit (Bluedent Smart) emitted a total energy of 16-24 J/cm^2^ (800-1200 mW/cm^2^ for 20 s). The temperature rise produced by QTH was significantly lower than that of LED for all test conditions, which was due to the lower power intensity leading to lower total energy.

This result was in accordance with the results of several studies. Guiraldo et al. [20] confirmed that the output power intensity and exposure time were the most important factors that influenced the temperature rise of dentin during composite curing. Summitt et al. [21] demonstrated that LED units caused a greater temperature rise in comparison with QTH. Durey et al. [22] studied the temperature rise within the dental pulp following the use of two LEDs and one QTH curing unit. The authors concluded that the pulpal temperature rose significantly more following the use of LED units compared to QTH units. On the other hand, our result disagreed with the work of Dogan et al. [23], who recorded a higher temperature rise using a QTH unit versus an LED system. However, this result might be due to the differences in the total energy of the QTH units used (30 J/cm^2^) compared to LED (11 J/cm^2^).

It was reported that there were significant chemical and morphological differences between permanent and primary teeth [9,24-27]. The dentin in primary teeth is less hard and durable compared to the dentin of permanent teeth. This is due to the lower concentrations of calcium and phosphorous in both peritubular and inter-tubular dentin in primary teeth compared to permanent teeth [28].

Johnson [10] indicated that the central area of the coronal dentin is considerably harder in permanent teeth than dentin from the same area in primary teeth. This is due to the greater degree of mineralization in permanent teeth compared to primary teeth. Therefore, it was expected that the temperature rise in primary dentin would be less pronounced than in permanent dentin. The latter was confirmed in this study, which found that regardless of dentin thickness or light curing unit used, the temperature rise in primary teeth was statistically significantly lower than in permanent teeth.

When applying resin composite in pediatric dentistry, it is important to select the correct photo-curing method that will not cause harmful overheating of the pulp. Although many studies have evaluated the effect of second-generation LEDs on temperature rise, there is a lack of studies comparing primary and permanent teeth. This study presented an in vitro experiment to measure the temperature rise accompanying the procedures of applying resin composite on primary and permanent teeth dentin with different thicknesses, but the current study has several limitations, such as blood microcirculation and the cellular and intercellular matrix of pulpal tissue, which acted as a refrigerant to heat [29]. Thus, the results cannot be applied directly to clinical conditions. Further studies should be performed to confirm the safety of the fast cure LED during the polymerization of resin composite and bonding agents.

## Conclusions

Dentin thickness is an important factor in protecting the pulp from possible thermal irritation. The temperature rise during polymerization of the resin composite with the second-generation LED appeared to be below 5.5 °C. Hence, it appears to be safe for use during the restoration of primary teeth. Primary teeth dentin might be more effective than permanent teeth dentin in protecting the dental pulp.
